# Near Infrared Optical Visualization of Epidermal Growth Factor Receptors Levels in COLO205 Colorectal Cell Line, Orthotopic Tumor in Mice and Human Biopsies

**DOI:** 10.3390/ijms140714669

**Published:** 2013-07-12

**Authors:** Gadi Cohen, Shimon Lecht, Mor Oron-Herman, Tatjana Momic, Aviram Nissan, Philip Lazarovici

**Affiliations:** 1 School of Pharmacy, Institute for Drug Research, Faculty of Medicine, the Hebrew University of Jerusalem, Jerusalem 91120, Israel; 2 Advanced Technology Center, the Chaim Sheba Medical Center, Tel-Hashomer 52621, Israel; 3 Department of Surgery, Hadassah-Hebrew University Medical Center, Jerusalem 24035, Israel

**Keywords:** COLO205 colorectal cell line, colorectal cancer biopsies, epidermal growth factor receptor, focal model, near infrared bio-imaging, orthotopic tumor

## Abstract

In this study, we present the applicability of imaging epidermal growth factor (EGF) receptor levels in preclinical models of COLO205 carcinoma cells *in vitro*, mice with orthotopic tumors and *ex vivo* colorectal tumor biopsies, using EGF-labeled with IRDye800CW (EGF-NIR). The near infrared (NIR) bio-imaging of COLO205 cultures indicated specific and selective binding, reflecting EGF receptors levels. *In vivo* imaging of tumors in mice showed that the highest signal/background ratio between tumor and adjacent tissue was achieved 48 hours post-injection. Dissected colorectal cancer tissues from different patients demonstrated *ex vivo* specific imaging using the NIR bio-imaging platform of the heterogeneous distributed EGF receptors. Moreover, in the adjacent gastrointestinal tissue of the same patients, which by Western blotting was demonstrated as EGF receptor negative, no labeling with EGF-NIR probe was detected. Present results support the concept of tumor imaging by measuring EGF receptor levels using EGF-NIR probe. This platform is advantageous for EGF receptor bio-imaging of the NCI-60 recommended panel of tumor cell lines including 6–9 colorectal cell lines, since it avoids radioactive probes and is appropriate for use in the clinical setting using NIR technologies in a real-time manner.

## 1. Introduction

In colorectal carcinoma (CRC), combination therapy using surgery, chemotherapy, monoclonal antibodies as well as radiotherapy are in common practice. However, novel emerging therapeutic and bio-imaging approaches are needed and expected to improve CRC diagnosis and treatment in order to personalize the targeted therapy with epidermal growth factor receptors (EGFR) monoclonal antibodies in BRAF-mutant metastatic patients [1]. An important characteristic of the diagnosis of CRC is differential high expression of EGFR, proposing these receptors as attractive targets for cancer diagnosis and treatment [2]. EGFR and its oncogenic forms such as HER-2/Erb-2 are frequently overexpressed in a variety of carcinoma tumors including CRC [3]. As a result, Cetuximab and Panitumumab, monoclonal antibody biological drugs, which block binding of EGF and transforming growth factor alpha (TGF-α) to EGFR have been frequently used in chemotherapy resistant CRC patients [1,4].

Recent studies have established the use of indocyanine green near infrared (NIR) dye for lymph node mapping in patients with cervical carcinoma [5,6]. It has also been used during intraoperative NIR imaging of breast carcinoma [7]. Since the interaction of indocyanine green with the tumor does not involve binding to a specific tumor marker and provides a high signal/background ratio (SBR), its use is of limited application in the diagnostic and intraoperative manipulations of CRC. In recent years, we [8] and others [9,10] described a novel NIR bio-imaging approach for CRC models using EGF-NIR which binds and visualize the heterogeneous expression of EGFR in CRC preclinical human tumor cell lines and clinical tissues [8].

Human tumor derived cell lines serve as basic preclinical models to study the biology of tumors, to investigate the therapeutic effects of anticancer drugs and for novel diagnostic purposes [11,12]. However, the high heterogeneity of tumors is only partially represented by a single cell line and therefore a panel of cell lines derived from the same tumor would be more likely to reflect its properties [12]. The National Cancer Institute (NIH) carefully characterized a large series of human cancer cell lines and established that 6–9 cell lines per tumor type (NCI-60 panel) would be sufficient to capture this heterogeneity [13,14]. For CRC, the most popular tumor derived cell lines, carefully analyzed for the expression of EGFR and chemotherapy response towards treatment with a typical immunotoxin [13], are represented by HT-29, HCT-116, SW-620, COLO205 and others [13,14]. In a previous study, we developed a near infrared bio-imaging approach of CRC models using HT-29, HCT-116 cells which express relatively high level of EGFR and the SW-620 cell line with low levels of this receptor expression [8]. To further establish this bio-imaging approach we focused in the present study on the cell line COLO205 which expresses low levels of EGFR and compared it to the human epithelial squamous carcinoma A431 cell line expressing high levels of EGFR as well as clinical samples, taken during tumor resection of colon cancer and normal colon tissues with heterogeneous expression of EGFR.

Therefore, the aim of the present study was to further extend and validate the proof of the concept that an EGF derived NIR diagnostic platform, could aid in determining the nature of preclinical CRC cell models and clinical tissues using NIR imaging.

We show that NIR images, intensity and the time dependence of dynamic NIR bio-imaging, could be used to characterize the level of EGFR expression in the COLO205 focal cell line model, in mice with orthotopic A431 tumors and in human CRC biopsies. This technology might be promising in approaches directed for a correlation of EGFR levels with other cellular properties in CRC cell lines and with CRC clinical outcomes.

## 2. Results

### 2.1. Validation of EGF-NIR Bio-Imaging Properties on A431 Carcinoma Cultures

**Figure 1:**
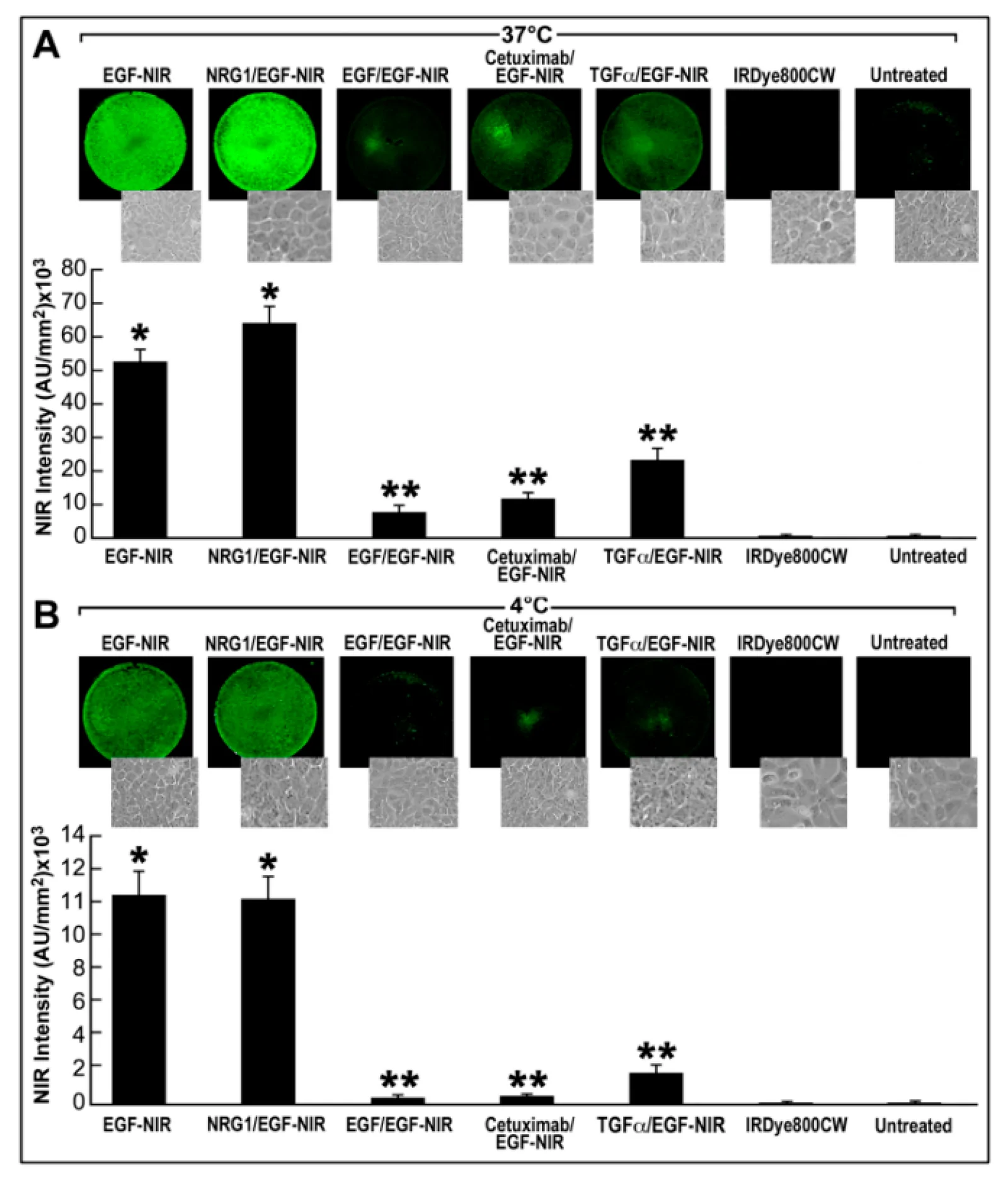
Specificity and selectivity of EGF-NIR probe measured by NIR bio-imaging of A431 cells; Monolayer of A431 cultures were incubated for 15 min at 4 °C (**A**) and 37 °C (**B**) with 7 nM EGF-NIR in the presence or absence of 100 nM unmodified EGF. Competition experiments with 500 nM of Cetuximab, TGF-α or NRG1 were also conducted. Nonspecific labeling of the cells was evaluated by incubating the cultures with 7 nM IRDye800CW. The NIR intensity was estimated (mean ± SD, *n* = 9) at the following conditions: resolution: 170–340 microns; pixel area: 0.03 mm^2^; quality: medium-low; focus offset: 3; channels: 800 nm; intensity: 1; Upper inserts: NIR scans; lower inserts: phase-contrast photomicrographs of the cultures, *p < 0.05 *vs.* IRDye800CW, **p < 0.05 *vs.* EGF-NIR.

Validation of EGF-NIR binding properties was performed using monolayers of A431 carcinoma cells expressing high levels of EGFR (Figure 1). In order to test EGF-NIR ligand-induced receptor internalization and cell surface binding, we compared NIR intensity signals of A431 cultures incubated with EGF-NIR in the presence or absence of different ligands at 37 °C (Figure 1A) and 4 °C (Figure 1B) conditions. The results show that binding of the EGF-NIR probe is specific and selective, resembling unmodified EGF, as evident from lack of competition with NRG1 and partial competition with EGF, TGF-α and Cetuximab, supporting our previous results with HT-29 CRC cell line [8].

**Figure 2:**
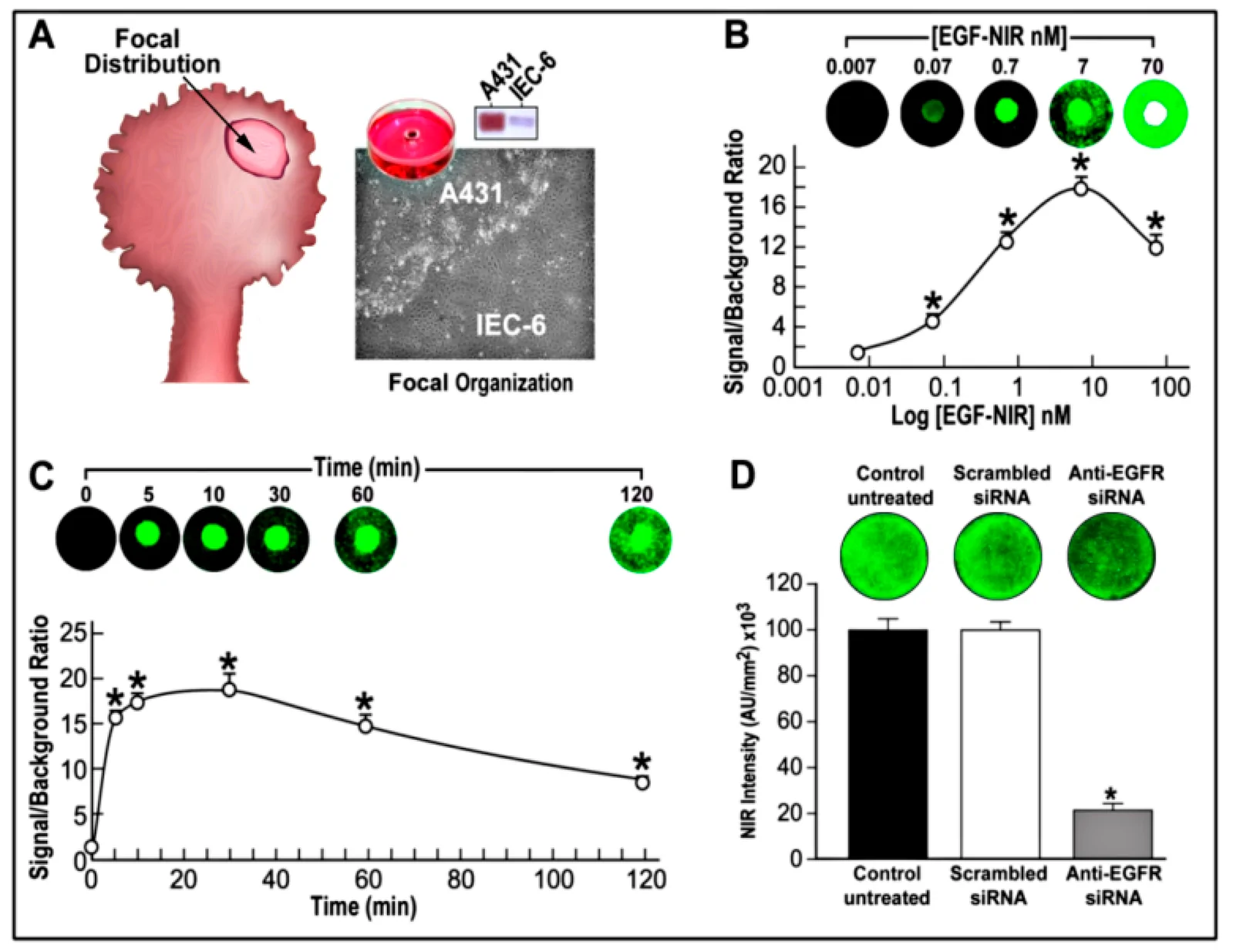
NIR bio-imaging of EGFR expression level in a focal model of A431 cells. (**A**) Left: CRC polyp scheme displaying high spots of transformed CRC cells (focal distribution); Right: focal plating (in a ring) of A431 monolayer surrounded by IEC6 enterocytes monolayer; Insert: Western blotting of EGFR protein in equal amounts of cell protein extract; (**B**) The relationship between the signal/background ratio (mean ± SD, *n* = 9) and EGF-NIR concentration. Insert: NIR scans; *p < 0.05 *vs.* 0.007 nM; (**C**) The kinetics of 7 nM EGF-NIR binding to the cell cultures (mean ± SD, *n* = 9); Insert: NIR scans; *p < 0.05 *vs.* 0 min; (**D**) siRNA induced knock down of EGFR measured by EGF-NIR bio-imaging. Homogenous monolayer cultures of A431 cells were transfected for 2 days with 5 nM anti EGFR Silencer select siRNA, or scrambled siRNA, or left untreated (control). Thereafter, EGFR expression was evaluated by NIR bio-imaging after incubating the cells with 7 nM EGF-NIR. The NIR imaging was estimated at the following conditions: resolution: 170–340 microns; pixel area: 0.03 mm^2^; quality: medium-low; focus offset: 3; channels: 800 nm; intensity: 1; *p < 0.05 *vs.* scrambled or control.

**Figure 3:**
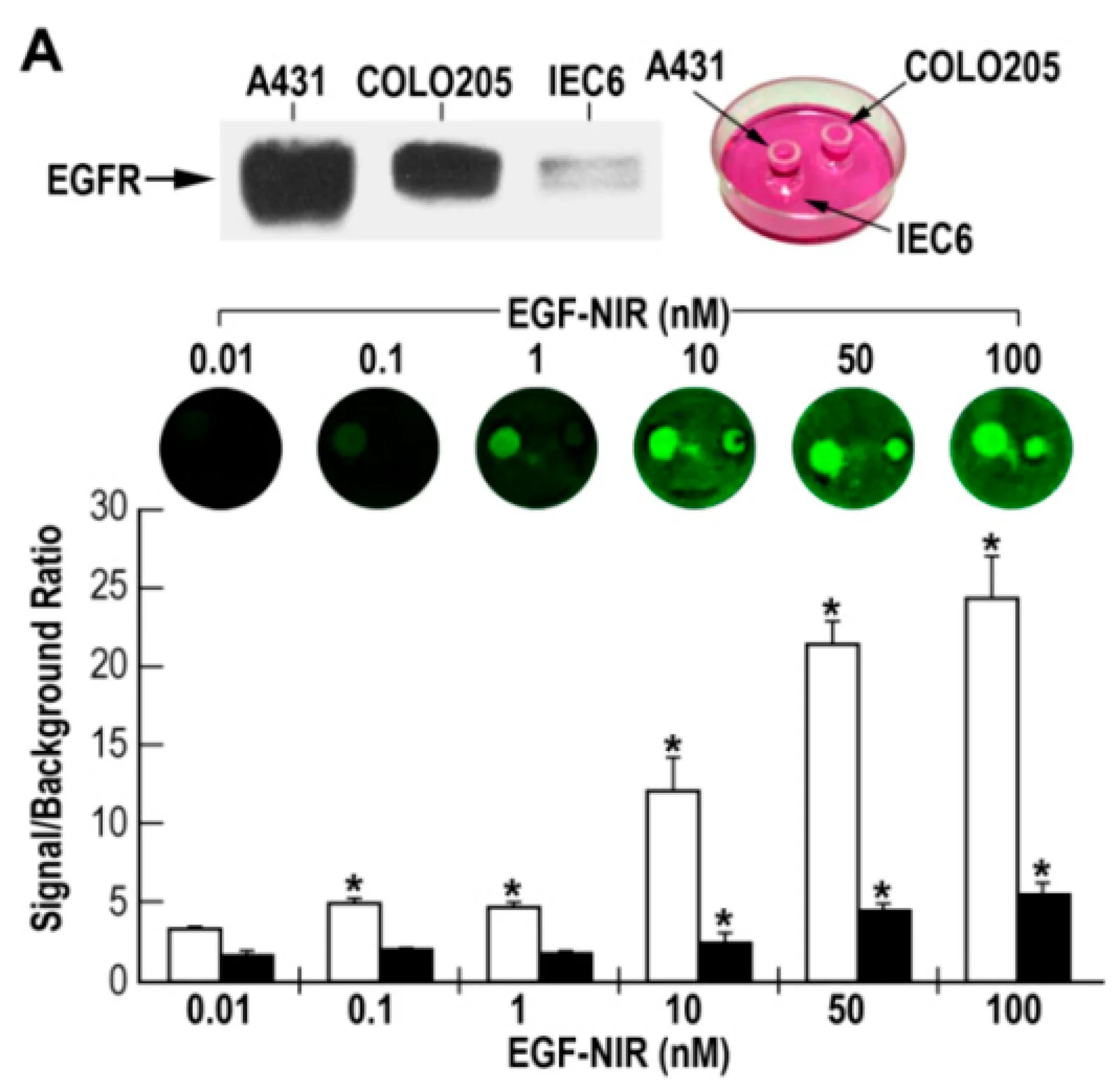

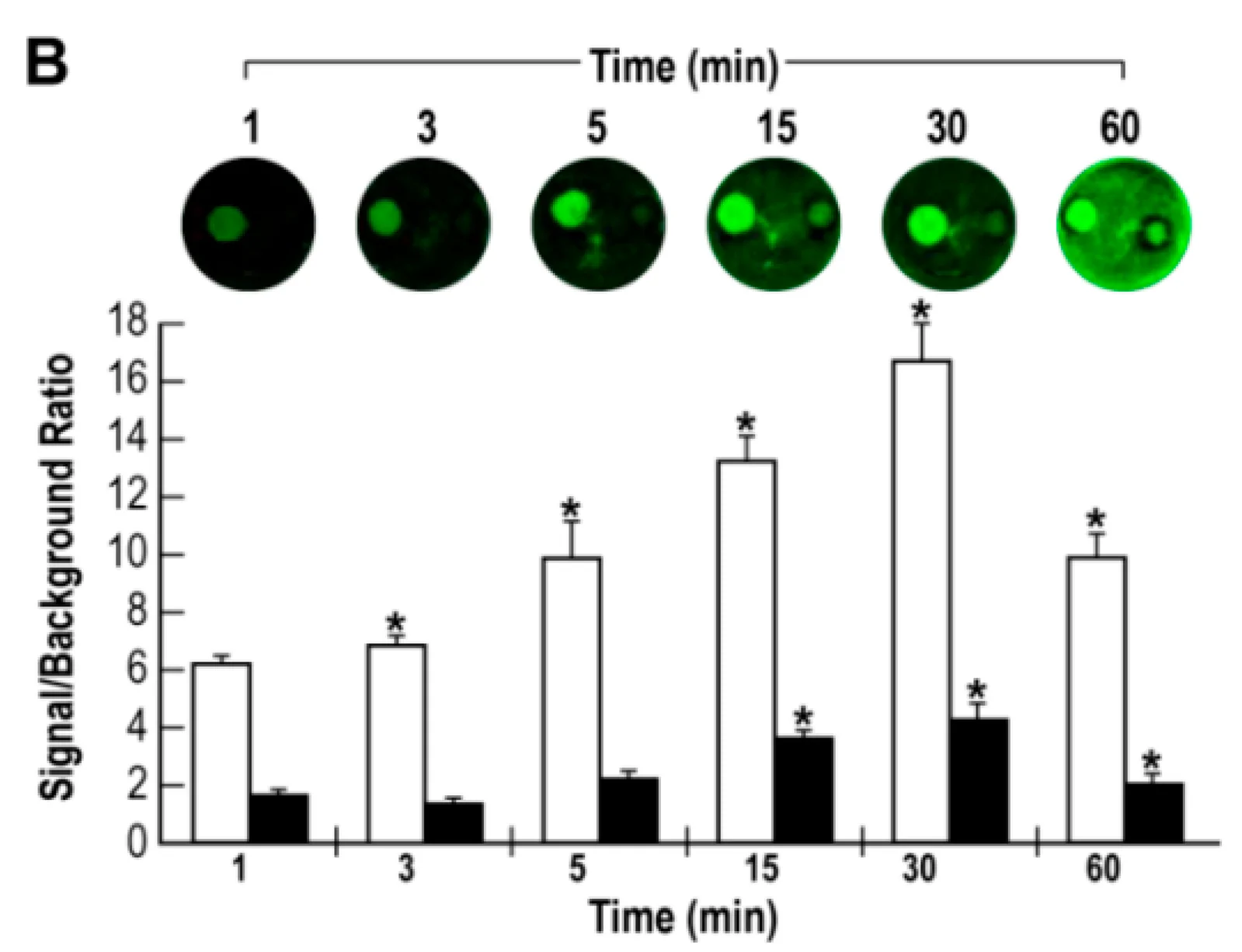
Saturation (**A**) and kinetics (**B**) of EGF-NIR binding to COLO205 CRC cells measured by NIR bio-imaging. Focal models of A431 (white bars) and COLO205 (black bars) cells were generated using the same plate, surrounded by IEC6 cells. The signal (CRC cell line) to background (IEC6) ratio was estimated at identical conditions for all cultures (mean ± SD, *n* = 9); The NIR imaging was estimated at the following conditions: resolution: 170–340 microns; pixel area: 0.03 mm^2^; quality: medium-low; focus offset: 3; channels: 800 nm; intensity: 1; (**A**) The cultures were incubated for 20 min at 37 °C with different concentration of EGF-NIR; Significance: *p < 0.05 *vs.* EGF-NIR value of 0.01 nM; Inserts: NIR scans; Upper insert: level of EGFR measured by Western blotting and focal organization of the cells; (**B**) The cultures were incubated for different periods of time at 37 °C with 7 nM EGF-NIR; *p < 0.05 *vs.* scrambled or control; Significance: *p < 0.05 *vs.* EGF-NIR value at time 1 min.

Since the distribution of EGFR in solid tumors is heterogeneous, we organized the carcinoma A431 cells in a focal configuration surrounded by non-transformed, rat small intestine epithelial IEC6 cells, as evident from the borderline between the A431 and IEC6 cells (Figure 2A). The Western blotting experiments demonstrate the high level of expression of EGFR in A431 cells compared to physiological levels expressed in IEC6 cells (Figure 2A-insert). Using this model, saturation (Figure 2B) and time-course (Figure 2C) binding experiments were performed. These experiments clearly indicate that the intensity of NIR signal (signal/background ratio) reflects the basic receptor binding properties: saturation upon the increase of ligand concentration and increased binding upon increasing duration of incubation. Based on these experiments, the optimal conditions for imaging with CRC derived COLO205 cells (Figure 3) were 20 min of incubation using 7 nM EGF-NIR. In another approach to demonstrate a direct relationship between EGFR levels, as detected by the NIR signal and EGF-NIR binding, we used small interference RNA (siRNA) in order to knock down EGFR expression in the cells. The efficiency of siRNA in decreasing EGFR mRNA levels was validated using RT-PCR and indicated a knock down of ~50%–70% (data not shown). EGFR protein levels, as evident from the EGF-NIR binding experiment, were reduced by ~80% (*p* < 0.05) as compared to untreated controls and cultures transfected with scrambled siRNA (Figure 2D). Therefore, we conclude that the decrease in NIR intensity measured (decreased specific binding of EGF-NIR) reflects the reduced expression of EGFR, in accordance with a previous study [8].

The results presented in Figures 1 and 2 validate the utilization of EGF-NIR for NIR bio-imaging, as previously reported [9,10].

### 2.2. NIR Bio-Imaging of EGF-NIR Binding to Preclinical Model of COLO205 Cultures

**Figure 4:**
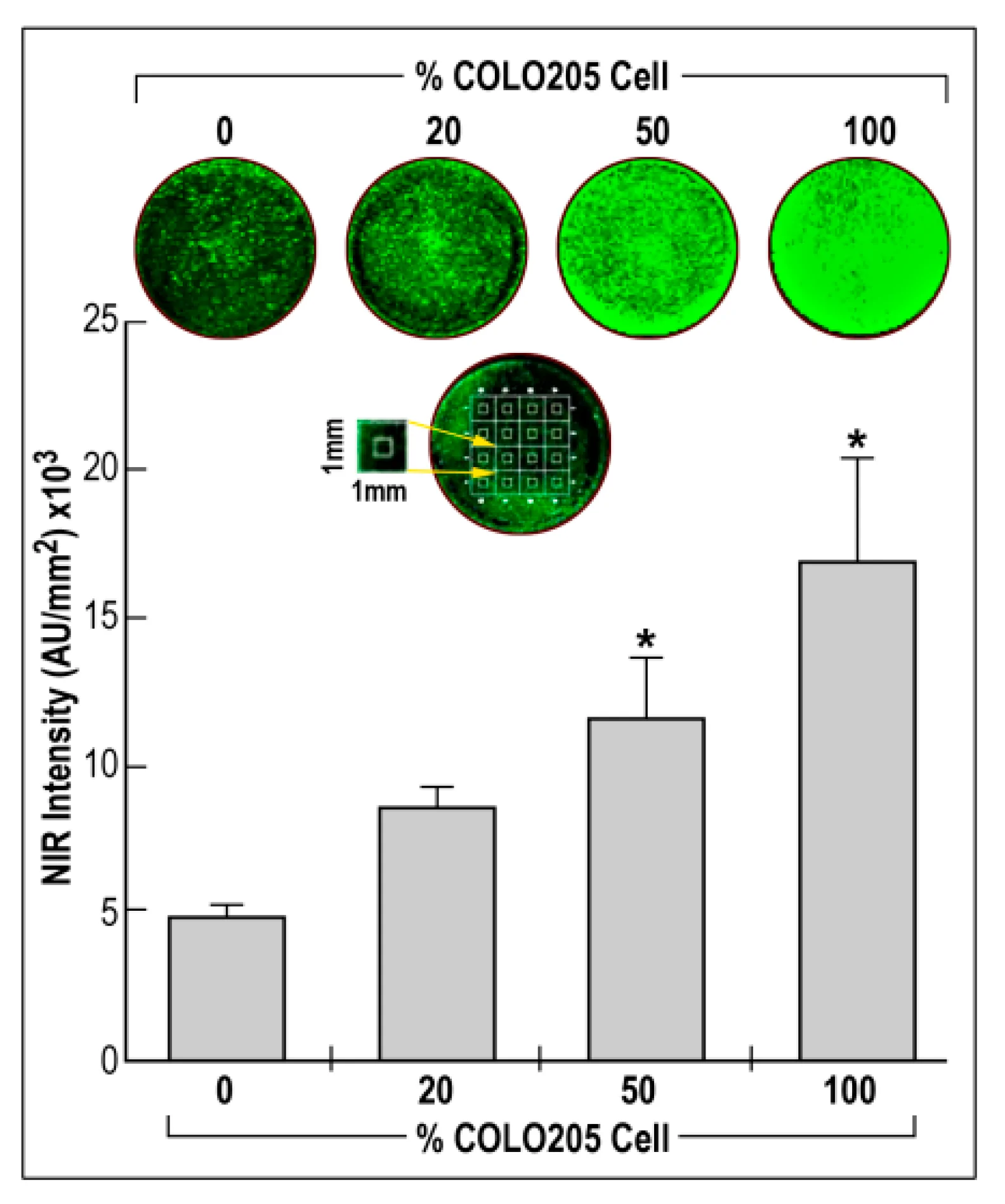
The relationship between NIR intensity and the number of EGFR positive carcinoma cells (COLO205) in a heterogeneous mixture with non-transformed cells (IEC6). 20 nM EGF-NIR was incubated for 20 min at 37 °C with a heterogeneous culture of both COLO205 and IEC6 cells at different complementary ratios (mean ± SD, *n* = 9); The NIR intensity was estimated at the following conditions: resolution: 170–340 microns; pixel area: 0.03 mm^2^; quality: medium-low; focus offset: 3; channels: 800 nm; intensity: 1; *p < 0.05 *vs.* scrambled or control. Significance: *p < 0.05 *vs.* 0% COLO205 cells. Insert: NIR scans; Lower insert: a photograph of a calibration scale suitable for endoscope with a resolution of 1 pixel (1 mm^2^).

Since EGFR-targeting therapies are currently in use for metastatic CRC [4], *in vitro* CRC preclinical models may be considered for NIR bio-imaging. To achieve this goal, we organized the COLO205 cell culture on a focal model of 4 mm diameter. This model may be suitable for optical coherence tomography, a clinical method that uses a larger diameter (5 mm) fiberoptic probe adjunct to colposcopy, for NIR detection of cervical intraepithelial neoplasia grade 2 or higher [15]. We utilized the COLO205 human CRC cell lines, which express moderate levels of EGFR, but higher levels compared to IEC6 cells [16]. For validation purposes A431 cells, were placed in rings surrounded by IEC6 cells. These experiments measured EGF-NIR binding, as would be viewed through an endoscope, and estimated the ratio between the signal of a carcinoma culture (COLO205, A431) to background binding (IEC6), in order to optically distinguish between tumor and normal tissues. The typical experiments presented in Figure 3 characterize saturation and kinetics of EGF-NIR binding to COLO205 and A431 cells. EGF-NIR saturated the receptors in both A431 cell cultures and in COLO205 cells; the maximal binding was at 30 min and thereafter decreased due to increased nonspecific binding to IEC6. The lower SBR in COLO205 compared to A431 in the different experiments is in direct correlation with the lower levels of EGFR in the cells (Figure 3A-insert). The SBR of EGF-NIR total binding to COLO205 cells was between 2 and 5 (4–5 fold lower then A431). The SBR values of nonspecific binding being 1, allowed calculations of specific binding at NIR intensity values of 1–4, which are in the sensitivity range of a NIR endoscope [17]. Furthermore, we found a direct relationship between the SBR and the number of EGFR positive carcinoma cells in a heterogeneous culture containing both COLO205 and IEC6 cells (Figure 4).

These findings propose that the 4 mm diameter focal model may be suitable for use *in vitro*, with NIR micro endoscopes [18], as the rigid Borescope with a 0 degree viewing angle and 1.5 mm outer diameter (Gradient Lens Corp., Rochester, NY, USA) commonly used for colonoscopies in clinical practice.

**Figure 5:**
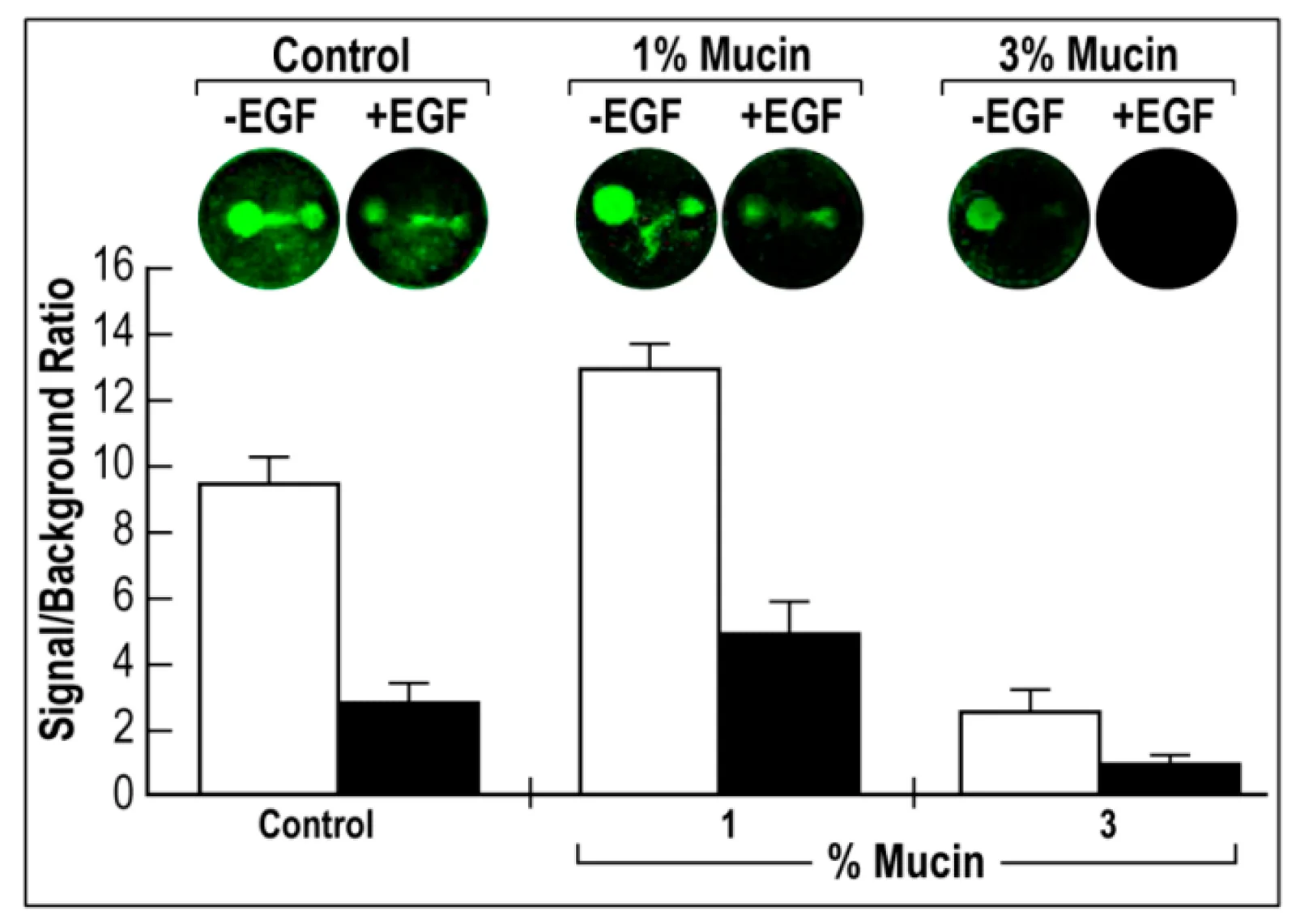
NIR bio-imaging of EGFR levels in focal cultures of A431/COLO205/IEC6 cells. 10 nM EGF-NIR was incubated for 15 min at 37 °C with the cultures pretreated with either 1% or 3% mucin, in the presence of 100 nM unlabeled EGF (+EGF) or in its absence (−EGF). SBR of specific binding was calculated for A431/IEC6 (white bars) and for COLO205/IEC6 (black bars). The upper inserts represent the cultures at the end of the experiment. Left focus—A431 cells; Right focus—COLO205 cells; NIR imaging was estimated (mean ± SD, *n* = 3) at the following conditions: resolution: 170–340 microns; pixel area: 0.03 mm^2^; quality: medium-low; focus offset: 3; channels: 800 nm; intensity: 1; *p < 0.05 *vs.* scrambled or control.

Adequate bowel preparation prior to colonic diagnostic procedures is essential to ensure proper visualization of the colon. Bowel preparation usually entails use of polyethylene glycol (PEG), sodium picosulphate or sodium phosphate (NaP), in combination with other prokinetics agents [19]. However, after this intensive preparation the luminal side of the colon, the intestine is still covered with the cytoprotective mucin layer, which may interfere with the ability of EGF-NIR to diffuse and bind to EGFR. To address this issue, we investigated the binding of EGF-NIR to a focal model, and a typical experiment investigating the effect of mucin on EGF-NIR binding is presented in Figure 5.

The results clearly indicate that the specific binding of EGF-NIR to both A431 and COLO205 cultures was slightly increased in the presence of 1% mucin and significantly decrease at 3% mucin. The physiological levels of mucin covering the luminal side of the colon are estimated to be in the range of 1–2 g/mL (0.1%–0.2%) [20]. Therefore the inhibitory effect of 3% mucin is not in the physiological range and the mucin is not expected to represent a significant barrier for the binding of EGF-NIR.

### 2.3. NIR Bio-Imaging of EGF-NIR Binding to Orthotopic A431 Tumors in Mice

**Figure 6:**
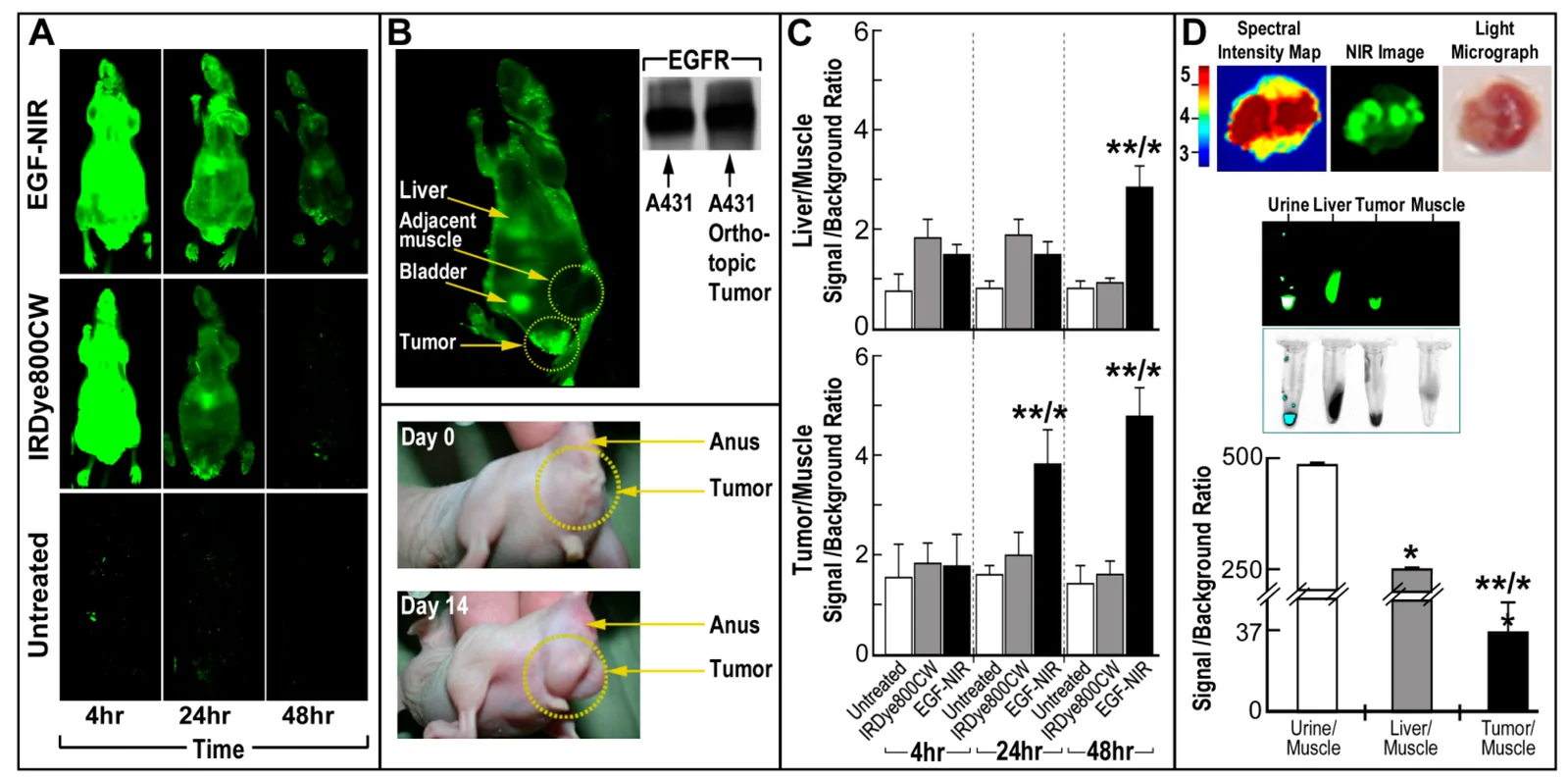
Whole body *in vivo* and isolated tissue *ex vivo* NIR bio-imaging of mice with A413 orthotopic tumors. (**A**) Time course of EGF-NIR accumulation in tissues of tumor bearing mice. The mice were injected intravenously with 1 nmol of EGF-NIR or IRDye800CW or left untreated (*n* = 3); (**B**) Tissue accumulation of EGF-NIR probes at 48 h; region of interest (ROI) marked with circles; Western blotting of tumor and A431 culture indicate EGFR expression; Right graphs: time course of EGF-NIR SBR in tumor and liver compared to skeletal muscle. Signal intensity at 800 nm was normalized to background fluorescence using an arbitrary tumor circle (ROI) compared to an identical area on the flank (adjacent muscle). Significance: *p < 0.05 compared to untreated; **p < 0.05 compared to mice injected with IRDye800CW; (**C**) EGF-NIR SBR in isolated tissue from the tumor-bearing mice 48 h after injection; (**D**) Upper three photos: NIR image, light micrograph and spectral intensity map of EGFR visualized by EGF-NIR (green and brown represent high levels of EGFR); NIR scanning of tissue samples in tubes, from mice 48 h after EGF-NIR injection. SBR between tissue of interest and skeletal muscle reflecting EGF-NIR distribution; NIR imaging was estimated at the following conditions: resolution: 170–340 microns; pixel area: 0.03 mm^2^; quality: medium-low; focus offset: 3; channels: 800 nm; intensity: 3; *p < 0.05 compared to muscle; **p < 0.05 compared to liver.

To confirm the suitability of EGF-NIR for *in vivo* NIR bio-imaging, orthotopic A431 tumors in nude mice were employed (Figure 6A,B). Three weeks after injection of A431 cell suspension into the mucosa, mice with visible tumors in the anal area (Figure 6B) were submitted for NIR bio-imaging. To confirm the expression of EGFR in tumors, western blotting analysis of dissected tumors was performed (Figure 6B). Figure 6A,B presents typical NIR fluorescence images of mice bearing EGFR positive tumors 4, 24 and 48 h after *i.v.* injection of 1 nmol EGF-NIR, an optimal dose which affords a high SBR, clearance and imaging results [9]. The control groups were injected with 1 nmol unconjugated IRDye800CW or left untreated. In the first four hours the animal injected with the imaging agent or dye show a very strong whole body fluorescence signal. After 24 h, about 80% of the fluorescence signal cleared, and after 48 h the signal intensity fell to background level, except in tissues expressing EGFR tumor, bladder and liver.

Quantitative two-dimensional surface measurements of SBRs 4, 24 and 48 h after EGF-NIR injection are plotted in Figure 6B. Each pixel (mm^2^) taken from the tumor and liver regions of interest (ROI) was compared to an identical area on the flank region (adjacent muscle). It is evident that after 24 h EGF-NIR specifically and significantly accumulated in the tumor, providing SBR value of approximately 4. This value is further increased at 48 h. In the liver, a tissue highly abundant in EGFR [21], a significant accumulation was observed after 48 h. We assume that the fast accumulation of EGF-NIR at 24 h reflects the very high levels of EGFR in the tumors. Estimations using macroscopic three-dimensional NIR imaging of harvested organs and urine, were performed 48 h after injection (Figure 6C). Urine showed a strong fluorescent NIR signal, since EGF is known to be excreted unmodified into the urine [22]. The liver strongly exceeded SBR NIR fluorescence compared to the tumor (Figure 6C). SBR of the isolated tumor compared to muscle tissue, was 37 ± 7.4, while the two dimensional SBR measurements of the whole animals was 4.8 ± 0.6. NIR bio-imaging of isolated organs is more sensitive than the non-invasive imaging of the organs scanning the whole animals, due to close proximity with the NIR imaging device and lack of absorbance and scattering of the NIR fluorescence by the animal tissues [9]. A spectral intensity map of the A431 tumor is presented in Figure 6C-upper part, indicating distinct areas expressing high levels of EGFR, which resemble CRC tumors.

### 2.4. Specificity of ex Vivo Bio-Imaging of EGF-NIR to Human CRC Biopsies

**Figure 7:**
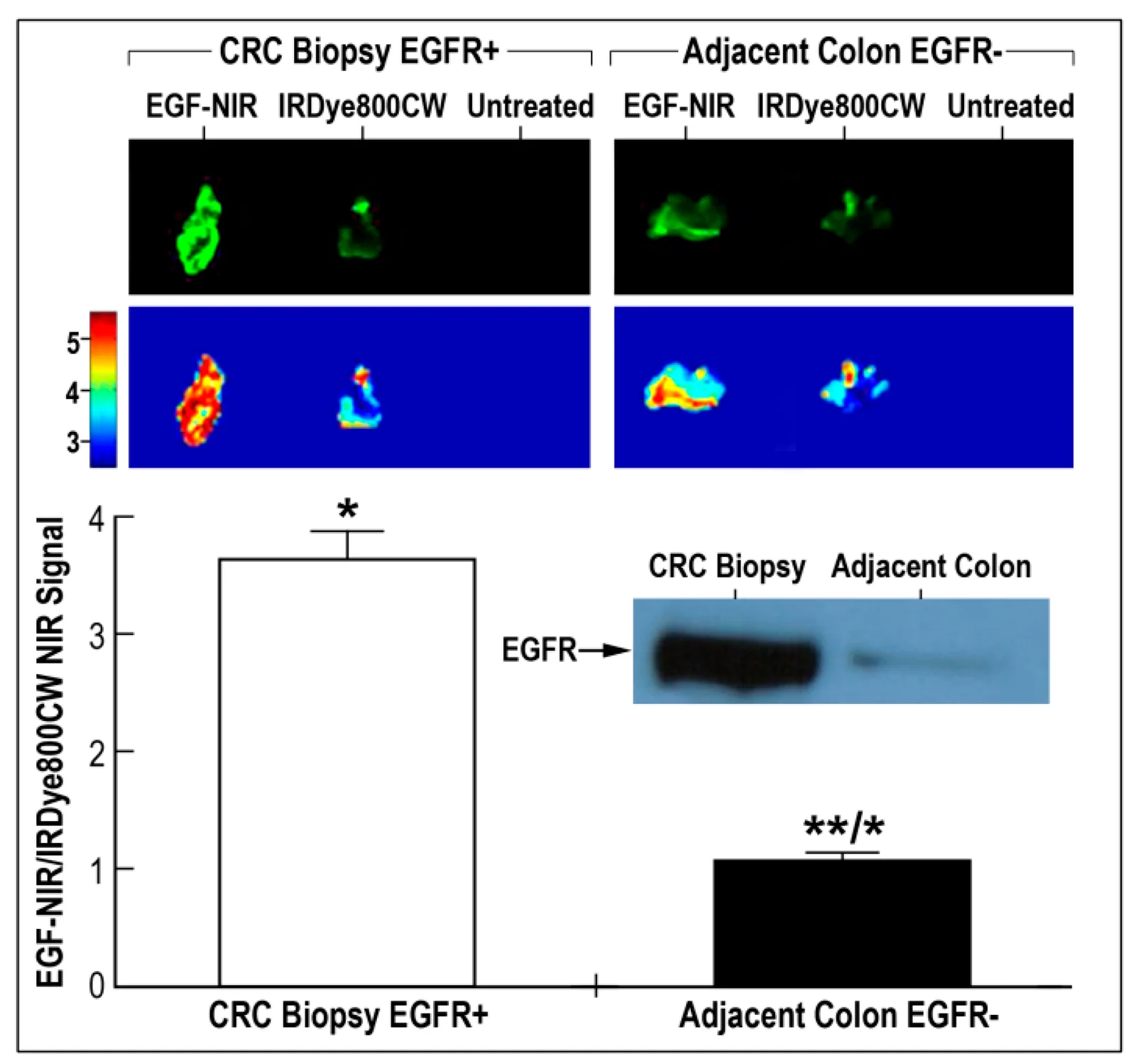
Bio-imaging of EGF-NIR binding to CRC tissues expressing high levels of EGFR, compared with adjacent colon tissues negative for EGFR expression. Slices (*n* = 4) of the different tissues (*n* = 19) were submitted for *ex vivo* binding assay of 45 min at 37 °C with 70 nM EGF-NIR. In control experiments, background fluorescence was measured in untreated slices and nonspecific absorption was measured in slices treated with 70 nM IRDye800CW. The NIR intensity was estimated at identical conditions for all slices (*n* = 76); NIR imaging was estimated at the following conditions: resolution: 170–340 microns; pixel area: 0.03 mm^2^; quality: medium-low; focus offset: 1; channels: 800 nm; intensity: 3; Significance: * *p* < 0.01 compared to IRDye800CW; ** *p* < 0.05 compared to CRC biopsy EGFR+. Upper inserts: NIR scans; Middle inserts: Western blotting for EGFR [Data originally published in Reference [8]].

Figure 7 presents *ex vivo* NIR bio-imaging of a relative large cohort of human CRC biopsies, performed with EGF-NIR. The binding experiments were conducted using tumor tissues slices identified by pathology examination as EGFR positive and slices of adjacent normal colonic mucosa tissues, located close to the tumor that were identified as EGFR negative. All slices were analyzed by Western blotting for EGFR protein expression levels (Figure 7—middle insert). The spectral maps of a typical slice incubated with either EGF-NIR (total binding), IRDye800CW (nonspecific absorption) or background fluorescence, are presented in the upper part of Figure 7. It is evident that the slices express little background fluorescence, IRDye800CW binding provides nonspecific absorption while EGF-NIR binding results in strong green fluorescence or a high intensity derived signal in brown, indicating a very high level of binding to the receptors. SBR quantitation between EGF-NIR and IRDye800CW NIR signals tested using CRC tissues that were found positive for EGFR (CRC biopsy EGFR+), reached high levels between 3 and 4. In the adjacent colon samples, the SBR between EGF-NIR and IRDye800CW was approximately 1. The higher mean value of the NIR signal in the CRC biopsy EGFR+ compared to the adjacent colon, strongly suggests that it is possible to generate a strong NIR fluorescence signal to detect EGFR positive CRC tumors. This could be utilized clinically using a NIR endoscope, as previously documented with an anti-human carcinoembryonic antigen (CEA) antibody labeled with indocyanine green (ICG) NIR dye [23].

## 3. Discussion

Colonoscopy followed by a biopsy and histopathological examination is the accepted gold standard for the diagnosis of CRC neoplastic lesions. Detection of adenomatous polyps is of utmost importance since the endoscopic resection of these neoplastic lesions have been shown to effectively prevent CRC [22]. Endoscopic assessment of colonic lesions or polyps is currently performed using endoscopal fiberoptic examination in the visible light spectrum. In cases of suspected flat polyps or areas of dysplasia in inflamed colonic tissue, fiberoptic colonoscopy is performed in conjunction with indigo carmine chromoscopy, to enhance mucosal contrast [24]. However, several clinical randomized control studies indicate variability in the sensitivity and specificity of the chromoscopic methods [25,26]. In recent years, NIR dyes were applied for bio-imaging of tumors in laboratory rodents and for imaging of superficial lesions such as axillary lymph nodes in humans [27,28]. Instruments, such as Zeiss Pentero, are currently available and several more are under development for clinical human imaging, therefore enabling bio-imaging of CRC tumors biomarkers with ligands labeled with NIR dyes or nanoparticles designed for bio-imaging [29].

We recently performed a study using the EGF-NIR bio-imaging agent prepared as described by Kovar *et al.*, evaluating its pharmacological properties for recognition of EGFR biomarkers in HT-29 CRC preclinical models [8]. In this present study, we further validate and extend the application of bio-imaging with the EGF-NIR platform to a new experimental model of CRC COLO205 cells compared to vulvar epithelium carcinoma A431 cells. Although, these two cell lines express low and high levels of EGFR expression, respectively, it was found that EGF-NIR binding and internalization was specifically and selectively visualized, independent of EGFR levels. The NIR fluorescent signal was proportional to the level of EGFR as indicated in binding experiments with cells expressing either low levels of EGFR, or in which EGFR was knocked down with siRNA. The EGF-NIR, rapidly (15–30 min) saturated (10 nM) the EGFR in COLO205 and A431 focal models as previously documented with HT-29 CRC cells [8]. It is important to stress that although HT-29 and COLO205 cells are popular in cancer research, in addition to their different EGFR expression, they also are heterogeneous in a variety of other cellular properties. COLO205 and HT-29 cells differ in their: cellular migration and metastatic abilities mediated by cyclooxygenase-2 [30,31]; proliferation response to insulin like growth factor [32]; sensitivity towards photosensitizers [33]; relative expression of glycosphingolipids tumor markers [34]; response to immunotoxins directed for glycosphingolipids antigens [35]; gene expression response to tyrosine kinase inhibitor [36,37] and non-steroidal anti-inflammatory drugs [38]; antitumor activities of different cytotoxic agents [39]; polymorphism of angiogenic factor receptors [40]; expression of different tumorigenic markers [41]; and many other properties. It will be important in future studies to address the relationship between low EGFR expression and the above different biological properties of these CRC derived tumor cell lines. EGF-NIR will clearly be an important tool to be considered for cell bio-imaging in addition to conventional western blotting and immunohistochemistry approaches. Present results on bio-imaging of EGFR with EGF-NIR in COLO205 further extend the applicability of this probe for estimation of the level of EGFR in CRC derived cell lines and are in accordance with a previous study in which cytotoxicity of EGFR-targeted immunoliposomes mediated specific and efficient drug delivery according to EGFR expression levels in COLO205 and HT-25 CRC and other cell lines [42].

A novel aspect addressed in the present study relates to the effect of mucin on the ability of EGF-NIR to visualize EGFR expression. For this purpose we investigated the effect of a high (3%) and low (1%) concentration of mucin on EGF-NIR binding to A431 cells, expressing high level of EGFR and COLO205 cells expressing low level of EGFR and which are also characterized by their ability to highly synthesize and secrete mucin and mucin like molecules [43]. Present results clearly indicate that in the presence of 1% mucin, EGF-NIR binding generated a high SBR, indicating that endoscopy evaluation with EGF-NIR may not be affected by residual mucin level on the intima of the intestine after bowel preparation.

Tissue localization of EGFR was analyzed using whole body NIR images of mice bearing A431 EGFR positive orthotopic tumors. Selective accumulation of EGF-NIR was measured 24 and 48 h after its injection, in the tumors as well as in EGFR positive organs, such as the liver. Quantization of EGF-NIR in tumors, normalized to skeletal muscle and compared to its level in the liver, indicated a faster accumulation of the agent in the tumor, compared to liver, suggestive of a specific EGFR mediated process. We calculated in the animal experiments and isolated tumor high SBRs of 4.8 ± 0.6 and 37 ± 7.4 for EGF-NIR binding, respectively. These values propose EGF-NIR as a suitable imaging agent for carcinoma visualization using NIR endoscopy, in which high SBRs are required. These findings are also reminiscent of pharmacokinetic experiments in which distribution of EGF conjugated to Cy5.5 fluorophore was measured in mice with breast cancer xenografts [21], with experiments of confocal endomicroscopy targeting EGFR with fluorescently labeled antibodies [44] and with our previous study using HT-29 orthotopic tumors visualized with EGF-NIR [8].

We further extended the utility of EGF-NIR for NIR imaging *ex vivo* using a larger cohort of human CRC biopsies. We demonstrated its binding to EGFR positive CRC biopsies but not EGFR negative adjacent colon tissue, as previously reported using EGF-NIR [8] or FITC-labeled anti-EGFR antibody [17]. The high spots of EGFR expression visualized in the different EGFR positive biopsies (Figure 7-insert) reflect the heterogeneous expression of the CRC tumor as previously reported [8]. Tumors are complex tissues that are characterized by profound spatial and temporal heterogeneity. Bio-imaging is central to their investigation because it can non-destructively and longitudinally characterize spatial variations in the tumor phenotype so that the system dynamics over time can be captured quantitatively [45] as demonstrated in the present study with EGF-NIR kinetics in cultures and orthotopic tumors. Present findings suggest that EGF-NIR may be a suitable platform technology for visualization of EGFR at different levels of expression in human biopsies of CRC tumors, complementary to the gold standard techniques of RT-PCR for EGFR mRNA quantification [46], *ex vivo* hybridization [47] and EGFR immunohistochemistry [26].

In conclusion, the EGF-NIR probe could be utilized for visualization of EGFR in the NCI-60 panel of tumor derived cell lines, and to complement other means of detection of adenomatous polyps and EGFR-expressing tumors in a clinical settings, either *ex vivo* or alternatively *in vivo*, with the aid of a suitable NIR endoscope. Additionally, the EGF-NIR probe may aid in selection of patients suitable for EGFR targeted therapy and in monitoring the therapeutic efficacy of anti-EGFR biological treatment.

## 4. Experimental Section

### 4.1. Cell Culture

Human colon carcinoma COLO205 and human epithelial squamous carcinoma A431 cell lines expressing high level of EGFR, and rat epithelial cell clone IEC6 with physiological levels of EGFR, were purchased from American Type Culture Collection (Manassas, VA, USA) and adjusted for growth in Dulbecco’s Modified Eagle’s Medium (DMEM) containing 10% fetal bovine serum, 2 mM L-glutamine, 10,000 U/mL penicillin and 100 μg/mL streptomycin. The cells were grown at 37 °C and 6% CO_2_ in a humidified incubator.

### 4.2. NIR Bio-Imaging of Monolayers of A431 Cells

A431 cells were plated at a density of 150,000 cells per well in 12 well tissue culture plates (Nunc, Rochester, NY, USA), generating a homogenous cell monolayer. In order to measure total binding, the culture medium was replaced with fresh medium containing 7 nM EGF-NIR (LI-COR Biosciences, Lincoln, NE, USA), for 15 min at 4 °C or 37 °C. Thereafter the cultures were washed three times with 1 mL phosphate buffered saline (PBS) and cell associated NIR intensity was estimated. In order to evaluate the nonspecific binding, sister cultures were incubated with the same concentration of EGF-NIR, in the presence of excess EGF of 100 nM. Specific binding by EGF-NIR imaging is defined as the difference between the NIR intensity of total binding and of nonspecific binding. Competition experiments with 500 nM of either Cetuximab, TGF-α or NRG1 were performed by concomitant incubation with EGF-NIR. The results are presented as the mean ± SD of at least three independent experiments (*n* = 9).

### 4.3. Preparation and NIR Bio-Imaging of Cells Organized in a Focal Model

In order to mimic a defined neoplastic CRC lesion surrounded by normal mucosa [16] and to enable direct measurements of SBR in the same experiment, a focal cell culture approach was used. 15 × 10^3^ A431 or COLO205 were confluently plated to two different 4 mm diameter cloning rings (Sigma-Aldrich, St. Louis, MO, USA). Cells were left to adhere for 2 h in an incubator, generating a polyp like area. Thereafter, 15 × 10^3^ IEC6 were plated in the cell-free area surrounding the cloning rings and left to adhere for 2 h at the same conditions generating a normal mucosa like area. At the end of the cell adherence step, the cloning rings were removed and the cells were washed with culture medium. Two days after generating the model, the cultures were subjected to binding and NIR imaging experiments as previously described [16]. SBR values indicate the ratio between the NIR fluorescent signal of the focal area (A431 or COLO205) and the NIR fluorescent signal of the outside area (IEC6).

### 4.4. NIR Bio-Imaging of Mucin Effect on EGF-NIR Binding

Mucin effect on EGF-NIR binding was examined on a focal cell culture approach. 10 nM EGF-NIR was incubated for 15 min at 37 °C with the cultures pretreated with either 1% or 3% mucin, in the presence or absence of 100 nM unlabeled EGF. SBR values indicate the ratio between the NIR fluorescent signal of the focal area (A431 or COLO205) and the NIR fluorescent signal of the outside area (IEC6).

### 4.5. Western Blotting of EGFR

The levels of EGFR in cell cultures were estimated upon extraction with cell lysis buffer (Cell Signaling Technology, Inc. Danvers, MA, USA). 50 μg protein lysates were separated by 10% SDS-PAGE and transferred to nitrocellulose membranes. Immunodetection was performed with monoclonal anti-EGFR antibody (Cell Signaling Technology, Inc. Danvers, MA, USA) followed by horseradish peroxidase (Jackson ImmunoResearch, West Grove, PA, USA) and developed with ECL (Pierce, Rockford, IL, USA) [8].

### 4.6. EGFR siRNA Silencing

To knock down EGFR, the standard amine transfection agent protocol of Ambion (Applied Biosystem, Austin, TX, USA) was followed. 5 nM of 21 mer anti-EGFR Silencer select siRNAs and scrambled siRNA were reverse transfected into A431 cell cultures using siPORTNeoFX transfection agent according to manufacturer protocol. The cells were applied on 12 well plates at a density of 80,000 cells/mL and were analyzed 3 days after transfections. Knock down of EGFR mRNA was confirmed by RT-PCR and evaluation of EGFR expression was performed by *in vitro* NIR imaging [8].

### 4.7. Preparation of Orthotopic Tumors Mice Model and NIR Bio-Imaging

Since orthotopic CRC tumors in mice models is challenging and not yet reported for the purpose of NIR imaging of A431 tumor in an epithelial mucosa tissue, we took advantage of a colorectal orthotopic A431 model. Briefly, male Balb/c nude (Harlan, Jerusalem, Israel) mice used in these experiments were maintained under the supervision and guidelines of The Chaim Sheba Medical Center Animal Committee. A431 cells were trypsinized, washed and resuspended at a concentration of 1 × 10^7^ cells/mL in PBS. For tumor implantation, mice were anesthetized by intra-peritoneal injection of a ketamine (100 mg/kg) and xylazine (20 mg/kg) mixture. Trans-anal injection of 1 × 10^6^ A431 cells was performed under microscope magnification (40×) using a 27 g needle. The injection was directed submucosally into the distal, posterior rectum, approximately 2–3 mm beyond the anal canal and into the rectal mucosa [48]. Mice were monitored two times weekly for tumor initiation and progression. Tumors reached a size of approximately 0.75 cm within 3–4 weeks.

*In vivo* NIR bio-imaging was performed at intervals of 1–8 h over a period of three days, using the LI-COR Biosciences small-animal imager Odyssey MousePOD^®^. During this procedure, all mice were starved overnight and anesthetized by intra-peritoneal injection of a ketamine (100 mg/kg) and xylazine (20 mg/kg) mixture. Mice were injected via the tail vein with either 1 nmol EGF-NIR (*n* = 5) or 1 nmol NIR-Dye (*n* = 3) or 0.9% saline (untreated, *n* = 3). Statistical analysis of the images for each mouse was normalized using the same intensity scales, under the conditions previously described. SBR was calculated using the following formula: SBR = (Mean NIR intensity of the tumor)/(Mean NIR intensity of adjacent muscle background). Regions of interest (ROI) with identical areas were used for both tumor and background. The standard deviation of mean backgrounds was calculated using 10–20 ROIs. Due to differences of tumor size among the animals receiving EGF-NIR, tumor signal was divided by a background signal of a similar size ROI. Two days post injection, tumors, skeletal muscle and liver tissues of euthanized mice were dissected and their urine samples were collected. The tissues were weighed, introduced into Ependorff plastic tubes and scanned on an Odyssey^®^ Infrared Imager. Isolated tissue SBR was generated by comparing the tissue accumulated EGF-NIR fluorescence signals to those of the adjacent muscle. The NIR imaging was estimated as described for cultures. Tumors images were processed by high resolution imaging [8] using applied spectral imaging software, Spectral ViewTM (ASI, Migdal Ha’Emek, Israel).

### 4.8. Human CRC Biopsies NIR Bio-Imaging

Patients with histologically confirmed primary adenocarcinoma of the colon were offered participation in the study. The study protocol was approved by the Institutional Review Board (IRB, Helsinki Committee) of Hadassah-Hebrew University Medical Center. All samples were obtained from consenting study subjects undergoing surgical tumor resection who signed a written informed consent. All specimens underwent routine macroscopic and microscopic analysis by a board certified pathologist according to the College of American Pathologists (CAP) guidelines of histopathology reporting (www.cap.org). Tissues identified by the study pathologist as colonic adenocarcinoma and adjacent normal tissues were then used for NIR bio-imaging. Fresh biopsies or adjacent normal colon tissues were divided in horizontal slices and incubated in ice-cold DMEM binding solution saturated with 95% O_2_ and 5% CO_2_[8]. The plates were maintained on ice for 45 min duration in DMEM and the binding experiment was performed by adding 70 nM EGF-NIR. The binding experiment was terminated by washing the tissue three times with cold PBS. Over a two year period 43 CRC biopsies and 23 adjacent colon tissue samples were evaluated. ROIs of identical size were used for the different experimental groups. The means and standard deviations of NIR intensity (arbitrary fluorescent units/mm^2^ area) were calculated using 10 ROIs in each individual slice. Each slice submitted for the EGF-NIR binding experiment was also evaluated after the imaging for EGFR expression by western blotting. The data achieved was categorized according to EGFR positive CRC biopsies, and adjacent colon tissues that were EGFR negative [8].

### 4.9. Statistics

All results are presented as the mean ± SD of large series of independent experiments using different batches of EGF, cells and mice tissues. All data was evaluated using the InStat3 statistics program (GraphPad, La Jolla, CA, USA). Statistically significant differences between experimental groups were determined by analysis of variance (ANOVA) with Bonferroni post-hoc test and considered significant when *p* < 0.05.

## 5. Conclusions

Our present study demonstrates the potential benefits of NIR optical imaging for CRC preclinical models and for clinical applications. Molecular imaging of EGFR using the EGF-NIR probe provides a potent, highly selective, non-invasive tool for the detection and characterization of EGFR-expressing tumors to be evaluated by either NIR endoscopes, scanners or other NIR modalities. This platform technology may complement immunohistochemistry, RT-PCR and other technologies, in measuring EGFR protein level in order to identify patients who are likely to benefit from anti-EGFR monoclonal antibody therapy.

## References

[1] Al-Marrawi M.Y., Saroya B.S., Brennan M.C., Yang Z., Dykes T.M., El-Deiry W.S. (2013). Off-label use of cetuximab plus sorafenib and panitumumab plus regorafenib to personalize therapy for a patient with V600E BRAF-mutant metastatic colon cancer. Cancer Biol. Ther.

[2] Shawver L.K., Slamon D., Ullrich A. (2002). Smart drugs: Tyrosine kinase inhibitors in cancer therapy. Cancer Cell.

[3] Bianco R., Gelardi T., Damiano V., Ciardiello F., Tortora G. (2007). Rational bases for the development of EGFR inhibitors for cancer treatment. Int. J. Biochem. Cell Biol.

[4] Italiano A. (2006). Targeting the epidermal growth factor receptor in colorectal cancer: Advances and controversies. Oncology.

[5] Crane L.M., Themelis G., Pleijhuis R.G., Harlaar N.J., Sarantopoulos A., Arts H.J., van der Zee A.G., Ntziachristos V., van Dam G.M. (2011). Intraoperative multispectral fluorescence imaging for the detection of the sentinel lymph node in cervical cancer: A novel concept. Mol. Imaging Biol.

[6] Van der Vorst J.R., Hutteman M., Gaarenstroom K.N., Peters A.A., Mieog J.S., Schaafsma B.E., Kuppen P.J., Frangioni J.V., van de Velde C.J., Vahrmeijer A.L. (2011). Optimization of near-infrared fluorescent sentinel lymph node mapping in cervical cancer patients. Int. J. Gynecol. Cancer.

[7] Troyan S.L., Kianzad V., Gibbs-Strauss S.L., Gioux S., Matsui A., Oketokoun R., Ngo L., Khamene A., Azar F., Frangioni J.V. (2009). The FLARE intraoperative near-infrared fluorescence imaging system: A first-in-human clinical trial in breast cancer sentinel lymph node mapping. Ann. Surg. Oncol.

[8] Cohen G., Lecht S., Arien-Zakay H., Ettinger K., Amsalem O., Oron-Herman M., Yavin E., Prus D., Benita S., Nissan A., Lazarovici P. (2012). Bio-imaging of colorectal cancer models using near infrared labeled epidermal growth factor. PLoS One.

[9] Kovar J.L., Volcheck W.M., Chen J., Simpson M.A. (2007). Purification method directly influences effectiveness of an epidermal growth factor-coupled targeting agent for noninvasive tumor detection in mice. Anal. Biochem.

[10] Kovar J.L., Johnson M.A., Volcheck W.M., Chen J., Simpson M.A. (2006). Hyaluronidase expression induces prostate tumor metastasis in an orthotopic mouse model. Am. J. Pathol.

[11] Sharma S.V., Haber D.A., Settleman J. (2010). Cell line-based platforms to evaluate the therapeutic efficacy of candidate anticancer agents. Nat. Rev. Cancer.

[12] Gillet J.P., Varma S., Gottesman M.M. (2013). The clinical relevance of cancer cell lines. J. Natl. Cancer Inst.

[13] Shoemaker R.H. (2006). The NCI60 human tumour cell line anticancer drug screen. Nat. Rev. Cancer.

[14] Yamori T. (2003). Panel of human cancer cell lines provides valuable database for drug discovery and bioinformatics. Cancer Chemother. Pharmacol.

[15] Liu Z., Belinson S.E., Li J., Yang B., Wulan N., Tresser N.J., Wang C., Mohr M., Zhang L., Zhou Y. (2010). Diagnostic efficacy of real-time optical coherence tomography in the management of preinvasive and invasive neoplasia of the uterine cervix. Int. J. Gynecol. Cancer.

[16] Wild R., Fager K., Flefleh C., Kan D., Inigo I., Castaneda S., Luo F., Camuso A., McGlinchey K., Rose W.C. (2006). Cetuximab preclinical antitumor activity (monotherapy and combination based) is not predicted by relative total or activated epidermal growth factor receptor tumor expression levels. Mol. Cancer Ther.

[17] Goetz M., Ziebart A., Foersch S., Vieth M., Waldner M.J., Delaney P., Galle P.R., Neurath M.F., Kiesslich R. (2010). *In vivo* molecular imaging of colorectal cancer with confocal endomicroscopy by targeting epidermal growth factor receptor. Gastroenterology.

[18] Rivera D.R., Brown C.M., Ouzounov D.G., Pavlova I., Kobat D., Webb W.W., Xu C. (2011). Compact and flexible raster scanning multiphoton endoscope capable of imaging unstained tissue. Proc. Natl. Acad. Sci. USA.

[19] Mathus-Vliegen E., Pellisé M., Heresbach D., Fischbach W., Dixon T., Belsey J., Parente F., Rio Tinto R., Brown A., Toth E. (2013). Consensus guidelines for the use of bowel preparation prior to colonic diagnostic procedures: Colonoscopy and small bowel video capsule endoscopy. Curr. Med. Res. Opin.

[20] Podolsky D.K., Isselbacher K.J. (1983). Composition of human colonic mucin. Selective alteration in inflammatory bowel disease. J. Clin. Invest.

[21] Ke S., Wen X., Gurfinkel M., Charnsangavej C., Wallace S., Sevick-Muraca E.M., Li C. (2003). Near-infrared optical imaging of epidermal growth factor receptor in breast cancer xenografts. Cancer Res.

[22] Hwang D.L., Lev-Ran A. (1990). Infusion of epidermal growth factor in mice: Organ distribution and urinary excretion. Regul. Pept.

[23] Ito S., Muguruma N., Kimura T., Yano H., Imoto Y., Okamoto K., Kaji M., Sano S., Nagao Y. (2006). Principle and clinical usefulness of the infrared fluorescence endoscopy. J. Med. Invest.

[24] Winawer S.J., Zauber A.G., Ho M.N., O’Brien M.J., Gottlieb L.S., Sternberg S.S., Waye J.D., Schapiro M., Bond J.H., Panish J.F. (1993). Prevention of colorectal cancer by colonoscopic polypectomy. N. Engl. J. Med.

[25] Pohl J., Lotterer E., Balzer C., Sackmann M., Schmidt K.D., Gossner L., Schaab C., Frieling T., Medve M., Mayer G. (2009). Computed virtual chromoendoscopy *versus* standard colonoscopy with targeted indigo carmine chromoscopy: A randomised multicentre trial. Gut.

[26] Le Rhun M., Coron E., Parlier D., Nguyen J.M., Canard J.M., Alamdari A., Sautereau D., Chaussade S., Galmiche J.P. (2006). High resolution colonoscopy with chromoscopy *versus* standard colonoscopy for the detection of colonic neoplasia: A randomized study. Clin. Gastroenterol. Hepatol.

[27] Funovics M.A., Alencar H., Su H.S., Khazaie K., Weissleder R., Mahmood U. (2003). Miniaturized multichannel near infrared endoscope for mouse imaging. Mol. Imaging.

[28] Shao X., Zheng W., Huang Z. (2011). Near-infrared autofluorescence spectroscopy for *in vivo* identification of hyperplastic and adenomatous polyps in the colon. Biosens. Bioelectron.

[29] Cohen G., Lazarovici P., Govil J.N. (2013). Molecular Imaging of Tumor Receptor and Signaling Molecules Using Near-Infrared Nano-Approches. Bioimaging, Biomaterials and Bioimaging of Nanotechnology Series.

[30] Leung E., McArthur D., Morris A., Williams N. (2008). Cyclooxygenase-2 inhibition prevents migration of colorectal cancer cells to extracellular matrix by down-regulation of matrix metalloproteinase-2 expression. Dis. Colon Rectum.

[31] Yamauchi T., Watanabe M., Hasegawa H., Nishibori H., Ishii Y., Tatematsu H., Yamamoto K., Kubota T., Kitajima M. (2003). The potential for a selective cyclooxygenase-2 inhibitor in the prevention of liver metastasis in human colorectal cancer. Anticancer Res.

[32] Michell N.P., Dent S., Langman M.J., Eggo M.C. (1997). Insulin-like growth factor binding proteins as mediators of IGF-I effects on colon cancer cell proliferation. Growth Factors.

[33] Chen Y.C., Lin J.K. (1996). Photodynamic anticancer agent merocyanine540 inhibits cell growth by apoptosis. Anticancer Res.

[34] Stroud M.R., Levery S.B., Nudelman E.D., Salyan M.E., Towell J.A., Roberts C.E., Watanabe M., Hakomori S. (1991). Extended type 1 chain glycosphingolipids: Dimeric Lea (III4V4Fuc2Lc6) as human tumor-associated antigen. J. Biol. Chem.

[35] Debinski W., Karlsson B., Lindholm L., Siegall C.B., Willingham M.C., FitzGerald D., Pastan I. (1992). Monoclonal antibody C242-Pseudomonas exotoxin A. A specific and potent immunotoxin with antitumor activity on a human colon cancer xenograft in nude mice. J. Clin. Invest.

[36] Morimoto A.M., Tan N., West K., McArthur G., Toner G.C., Manning W.C., Smolich B.D., Cherrington J.M. (2004). Gene expression profiling of human colon xenograft tumors following treatment with SU11248, a multitargeted tyrosine kinase inhibitor. Oncogene.

[37] Roberts W.G., Whalen P.M., Soderstrom E., Moraski G., Lyssikatos J.P., Wang H.F., Cooper B., Baker D.A., Savage D., Dalvie D. (2005). Antiangiogenic and antitumor activity of a selective PDGFR tyrosine kinase inhibitor, CP-673,451. Cancer Res.

[38] He H., Xia H.H., Wang J.D., Gu Q., Lin M.C., Zou B., Lam S.K., Chan A.O., Yuen M.F., Kung H.F. (2006). Inhibition of human telomerase reverse transcriptase by nonsteroidal antiinflammatory drugs in colon carcinoma. Cancer.

[39] Yamaguchi T., Kakefuda R., Tajima N., Sowa Y., Sakai T. (2011). Antitumor activities of JTP-74057 (GSK1120212), a novel MEK1/2 inhibitor, on colorectal cancer cell lines *in vitro* and *in vivo*. Int. J. Oncol.

[40] Estevez-Garcia P., Castaño A., Martin A.C., Lopez-Rios F., Iglesias J., Muñoz-Galván S., Lopez-Calderero I., Molina-Pinelo S., Pastor M.D., Carnero A. (2012). PDGFRα/β and VEGFR2 polymorphisms in colorectal cancer: Incidence and implications in clinical outcome. BMC Cancer.

[41] Wang L.B., Zheng S., Zhang S.Z., Peng J.P., Ye F., Fang S.C., Wu J.M. (2005). Expression of ST13 in colorectal cancer and adjacent normal tissues. World J. Gastroenterol.

[42] Mamot C., Ritschard R., Küng W., Park J.W., Herrmann R., Rochlitz C.F. (2006). EGFR-targeted immunoliposomes derived from the monoclonal antibody EMD72000 mediate specific and efficient drug delivery to a variety of colorectal cancer cells. J. Drug Target.

[43] Sikut R., Sikut A., Zhang K., Baeckström D., Hansson G.C. (1998). Reactivity of antibodies with highly glycosylated MUC1 mucins from colon carcinoma cells and bile. Tumor Biol.

[44] Mahmood U. (2010). Optical molecular imaging approaches in colorectal cancer. Gastroenterology.

[45] Gillies R.J., Anderson A.R., Gatenby R.A., Morse D.L. (2010). The biology underlying molecular imaging in oncology: From genome to anatome and back again. Clin. Radiol.

[46] Ho-Pun-Cheung A., Bascoul-Mollevi C., Assenat E., Boissière-Michot F., Bibeau F., Cellier D., Ychou M., Lopez-Crapez E. (2009). Reverse transcription-quantitative polymerase chain reaction: Description of a RIN-based algorithm for accurate data normalization. BMC Mol. Biol.

[47] Patel V.G., Shum-Siu A., Heniford B.W., Wieman T.J., Hendler F.J. (1994). Detection of epidermal growth factor receptor mRNA in tissue sections from biopsy specimens using *in situ* polymerase chain reaction. Am. J. Pathol.

[48] Donigan M., Norcross L.S., Aversa J., Colon J., Smith J., Madero-Visbal R., Li S., McCollum N., Ferrara A., Gallagher J.T. (2009). Novel murine model for colon cancer: Non-operative trans-anal rectal injection. J. Surg. Res.

