# Correction: Cohen et al. Near Infrared Optical Visualization of Epidermal Growth Factor Receptors Levels in COLO205 Colorectal Cell Line, Orthotopic Tumor in Mice and Human Biopsies. *Int. J. Mol. Sci.* 2013, *14*, 14669–14688

**DOI:** 10.3390/ijms27135893

**Published:** 2026-06-30

**Authors:** Gadi Cohen, Shimon Lecht, Mor Oron-Herman, Tatjana Momic, Aviram Nissan, Philip Lazarovici

**Affiliations:** 1 School of Pharmacy, Institute for Drug Research, Faculty of Medicine, The Hebrew University of Jerusalem, Jerusalem 91120, Israel; 2 Advanced Technology Center, The Chaim Sheba Medical Center, Tel-Hashomer 52621, Israel; 3 Department of Surgery, Hadassah-Hebrew University Medical Center, Jerusalem 24035, Israel

In the original publication [1], there was a mistake in the legend for Figure 7. Figure 7 presented a Western blot insert previously published in reference 8 PLOS ONE (Cohen G, Lecht S, Arien-Zakay H, Ettinger K, Amsalem O, et al. (2012) Bio-Imaging of Colorectal Cancer Models Using Near Infrared Labeled Epidermal Growth Factor. PLoS ONE 7(11): e48803. doi:10.1371/journal.pone.0048803). This insert should have been identified as being from that source.

The correct legend appears below:

**Figure 7.** Bio-imaging of EGF-NIR binding to CRC tissues expressing high levels of EGFR, compared with adjacent colon tissues negative for EGFR expression. Slices (*n* = 4) of the different tissues (*n* = 19) were submitted for ex vivo binding assay of 45 min at 37 °C with 70 nM EGF-NIR. In control experiments, background fluorescence was measured in untreated slices and nonspecific absorption was measured in slices treated with 70 nM IRDye800CW. The NIR intensity was estimated at identical conditions for all slices (*n* = 76); NIR imaging was estimated at the following conditions: resolution: 170–340 microns; pixel area: 0.03 mm^2^; quality: medium-low; focus offset: 1; channels: 800 nm; intensity: 3; Significance: * *p* < 0.01 compared to IRDye800CW; ** *p* < 0.05 compared to CRC biopsy EGFR+. Upper inserts: NIR scans; Middle inserts: Western blotting for EGFR [Data originally published in Reference [8]].

The authors state that this correction does not affect the scientific results or conclusions of the paper. This correction was approved by the Academic Editor. The original publication has also been updated.
